# Sequence and Annotation of the Apicoplast Genome of the Human Pathogen *Babesia microti*

**DOI:** 10.1371/journal.pone.0107939

**Published:** 2014-10-03

**Authors:** Aprajita Garg, Anna Stein, William Zhao, Ankit Dwivedi, Roger Frutos, Emmanuel Cornillot, Choukri Ben Mamoun

**Affiliations:** 1 Department of Internal Medicine, Section of Infectious Diseases, Yale School of Medicine, New Haven, Connecticut, United States of America; 2 Centre d'étude d'agents Pathogènes et Biotechnologies pour la Santé - UMR 5236, Institut de Biologie Computationnelle, Montpellier, France; 3 CIRAD, UMR 17, Cirad-Ird, TA-A17/G, Campus International de Baillarguet, Montpellier, France; Obihiro University of Agriculture and Veterinary Medicine, Japan

## Abstract

The apicomplexan intraerythrocytic parasite *Babesia microti* is an emerging human pathogen and the primary cause of human babesiosis, a malaria-like illness endemic in the United States. The pathogen is transmitted to humans by the tick vector, *Ixodes scapularis*, and by transfusion of blood from asymptomatic *B. microti*-infected donors. Whereas the nuclear and mitochondrial genomes of this parasite have been sequenced, assembled and annotated, its apicoplast genome remained incomplete, mainly due to its low representation and high A+T content. Here we report the complete sequence and annotation of the apicoplast genome of the *B. microti* R1 isolate. The genome consists of a 28.7 kb circular molecule encoding primarily functions important for maintenance of the apicoplast DNA, transcription, translation and maturation of organellar proteins. Genome analysis and annotation revealed a unique gene structure and organization of the *B. microti* apicoplast genome and suggest that all metabolic and non-housekeeping functions in this organelle are nuclear-encoded. *B. microti* apicoplast functions are significantly different from those of the host, suggesting that they might be useful as targets for development of potent and safe therapies for the treatment of human babesiosis.

## Introduction

Human babesiosis is an emerging infectious disease caused by a select group of intraerythrocytic protozoan parasites defining a new clade in the Apicomplexan phylum distinct from those encompassing *Plasmodium* species, *Theileria* species or *Babesia bovis* [1]. Most babesiosis infections in humans are caused by *Babesia microti* and are transmitted by *Ixodes* ticks, the vectors responsible for transmission of several other pathogens including *Borrelia*, *Anaplasma* and *Ehrlichia* species. Human babesiosis is endemic in northeastern and northern midwestern United States but has also been reported in Europe, Asia, Africa, Australia and South America [2], [3]. Depending on their immune status and age, patients with human babesiosis can experience mild, moderate or severe illness with the latter possibly leading to multi-organ system failure and death. In the United States, the mortality rate approaches 9% in hospitalized patients and ∼20% in immunocompromised hosts [3], [4]. As a result babesiosis has been recognized as an emerging health threat [5], and since 2011 has been designated as a nationally notifiable disease by the Center for Disease Control [6].

The ability of *B. microti* to invade and multiply within human red blood cells, and the lack of effective tools for large-scale screening of blood for *B. microti* infection from asymptomatic donors make this parasite a major risk to the national blood supply [7], [8]. Accordingly, *B. microti* is now considered the most commonly reported transfusion-transmitted pathogen in the United States [5], and the number of documented cases of acquired infections by transfusion has substantially increased over the years [9]. Although babesiosis therapy, which consists of combination of atovaquone and azithromycin or clindamycin and quinine [10], is considered generally effective, adverse events and disease failure and relapse can occur in some patients.

Recent efforts aimed to probe the diversity, pathogenicity and metabolism of *B. microti* and to identify new markers and targets for diagnosis and therapy of human babesiosis have led to the completion of the first genomic sequence of a clinical clone named R1 [11]. Subsequently Whole Genome maps of two *B. microti* strains R1 and Gray were reported [12]. These genomic analyses revealed that the genome of *B. microti* is less than 7Mbp, making it the smallest nuclear genome among apicomplexa [11]. Phylogenetic analyses placed *B. microti* in a new lineage among apicomplexan parasites distinct from *B. bovis* and *Theileria* species [11]. The genome effort has also revealed that the parasite has two DNA-containing organelles, the mitochondria and the apicoplast. The apicoplast is a non-photosynthetic plastid that plays an essential role in parasite development and survival [13], [14]. Genetic and biochemical studies in malaria and related parasites have shown that this organelle hosts important metabolic and housekeeping processes, which are critical for parasite survival within host cells (reviewed in [15]). Some of these pathways exist in *B. microti* and are significantly different from their host counterparts thereby offering new opportunities for the development of selective therapies for treatment of human babesiosis [11].

While the linear mitochondrial genome of *B. microti* has been fully characterized by two independent studies [12], [16], the apicoplast genome of this parasite remained partially assembled due to its high A+T content and to its low representation. Here we report the completion of the genomic sequence of the apicoplast of the *B. microti* R1 isolate. We show that this genome consists of a 28.7 kb circular molecule, which encodes genes involved in maintenance of the apicoplast DNA, transcription, translation and maturation of organellar proteins. Sequence analysis of the *B. microti* apicoplast genome and genome comparisons revealed that major gene alterations and rearrangements occurred in the apicoplast genomes during the evolution of piroplasms.

## Materials and Methods

### Sequencing, assembly and annotation of the apicoplast genome

Genomic DNA used to complete the sequence of the R1 apicoplast genome was previously described [11], [12]. The apicoplast genome sequence was obtained by primer walking using long PCR reactions. PCR amplification was performed using total genomic DNA and primers designed following sequencing of apicoplast genome contigs obtained after the first genome assembly [11]. Sequencing was performed on both strands and from at least two PCR products using specific primer pairs (**Table S1 in File S1**). The complete sequence of the apicoplast genome was deposited in the European Nucleotide Archive with Accession Number LK028575. Genome annotation was performed using Artemis (https://www.sanger.ac.uk/resources/software/artemis/) [17] and BLAST (http://blast.ncbi.nlm.nih.gov/Blast.cgi). Prediction of tRNA genes was performed using tRNAscanSE 1.21 (http://lowelab.ucsc.edu/tRNAscan-SE/) with the following parameters: search mode: “default”, and source: “Mito/Chloroplast” [18]. Genetic maps were obtained using CGView (http://stothard.afns.ualberta.ca/cgview_server) [19] and GenomeVx (http://wolfe.ucd.ie/GenomeVx) [19]. Genome comparisons were performed using Mauve (http://gel.ahabs.wisc.edu/mauve) [20] and edited using ACT (https://www.sanger.ac.uk/resources/software/act) [21]. Manual editing to correct the above automated analyses was completed on a gene-by-gene basis as needed. The following entries were used for comparative analyses: AAXT01000007 (*B. Bovis*), HM222968 (*Chromera*), fusion of X95275 (IRA) and X95276 (IRB) (*P. falciparum*), AAGK01000009 (*T. parva*), U87145 (*T. gondii*). Phylogenetic analyses were performed using Phylogeny.fr with default options [22]. TMpred (http://www.ch.embnet.org/software/TMPRED_form.html) and Pfam (http://pfam.sanger.ac.uk/) were used for transmembrane domain and motif predictions [23], [24].

## Results and Discussion

### Sequence analysis of the circular apicoplast genome of *B. microti*

**Figure 1:**
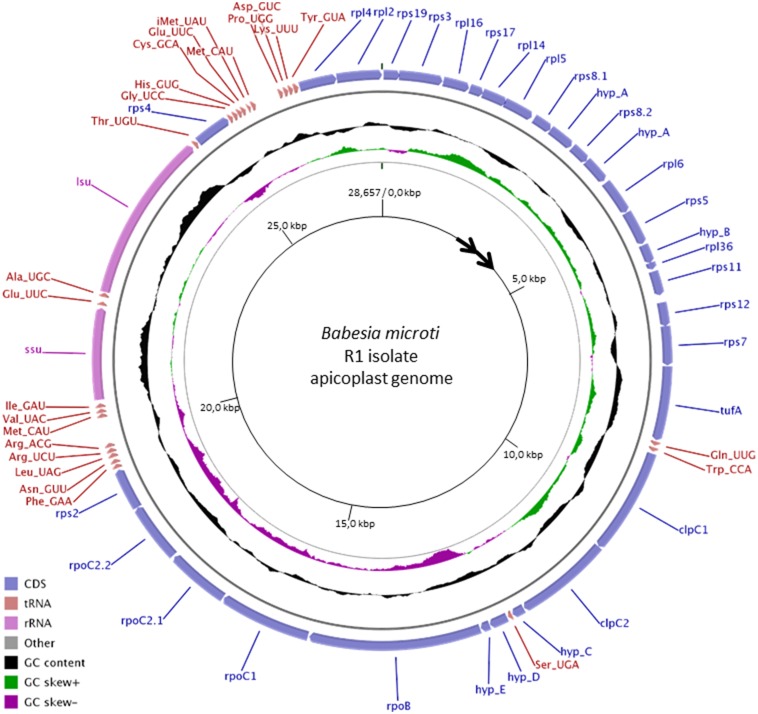
Graphical circular map of the apicoplast genome of *B. microti* R1 isolate. The map was designed using CGview and GenomeVx. From outside to center: coding sequence (CDS), % G+C, GC skew and base coordinates. *hypA-E* refer to five hypothetical protein encoding genes found in the apicoplast genome of *B. microti*.

Previous efforts aimed to sequence the nuclear and organellar genomes of *B. microti* identified several contigs representing partial sequences of the apicoplast genome of this parasite. In order to generate a complete sequence of the apicoplast genome, we performed primer walking using total genomic DNA and specific primers derived from the complete sequencing of the individual contigs. Sequencing and assembly revealed that the apicoplast genome of *B. microti* is composed of a circular molecule of 28.7 kbp. It is the smallest apicoplast genome found in apicomplexan parasites. The genome is 86% A+T rich and has a coding density of over 98%. It encodes SSU and LSU rRNAs, 18 ribosomal proteins, an EF-Tu elongation factor, three subunits of the RNA polymerase, 2 copies of the ClpC chaperone, 24 tRNAs and five hypothetical proteins ranging in size between 49 (Hyp-E) and 140 amino acids (Hyp-A) (Fig. 1). All genes encoded in the apicoplast genome of *B. microti* are transcribed in the same orientation (Fig. 1). AUG or AUA serve as initiators for 18 and 13 of the 31 codon sequences (CDSs), respectively, and are preceded by an A-rich region that may play an important role in the recruitment of the ribosome. Consistent with this unusual translation initiation, a single Met-itRNA with an UAU anticodon was found in the apicoplast genome. Termination codons used in the CDSs of apicoplast genome include UAA (25 of the 31 CDSs) and UGA (6 of the 31 CDSs). This finding is consistent with the annotation of the *B. microti* nuclear genome, which identified an apicoplast targeting motif in the RF2 release factor [11]. No UAG stop codons were found in the apicoplast genome of *B. microti* consistent with the lack of an apicoplast targeting sequence in the RF1 release factor encoded by the nuclear genome [11].

The majority of CDSs in the apicoplast genome of *B. microti* do not overlap, and only four coding sequences were found to overlap over one to three codons, making the start of one CDS part of the stop codon of the previous CDS. tRNAscanSE analyses identified 24 tRNAs. In addition to the Met-itRNA, other tRNAs with a U at the first position of the anticodon have been found but the Wobble pairing U - G represents less than 1% of codons in the *B. microti* apicoplast genome. Half of the tRNAs known to decode codons ending with a U or a C were not detected by tRNAscanSE; these tRNAs might have a sequence that is too divergent from others to be recognized by tRNAscanSE. Similar to *P. falciparum*, CGU is the only codon found in the *B. microti* apicoplast genome used for arginine in the CGN group.

Detailed analysis of the ribosomal proteins and RNAs predicted from the *B. microti* apicoplast genome revealed significant similarity with other apicomplexa. The genome encodes proteins of the small (11 rps proteins) and large (7 rpl proteins) ribosomal subunits. Additional ribosomal proteins are encoded by the nuclear genome and targeted to the apicoplast [11]. Association of nuclear and apicoplast encoded ribosomal proteins with 16S- and 23S-like rRNA molecules may form the apiRibosome of *B. microti*. No 5S ribosomal RNA-encoding *rff* gene could be found in the *B. microti* apicoplast genome. This finding suggests that the apicoplast ribosomes of *B. microti* are independent of 5S rRNA or that the apicoplast can either import 5S RNA from the cytoplasm, as was previously shown for mammalian mitochondria [25], [26], or expresses a gene with a sequence highly divergent from known *rff* genes. Noteworthy, whereas the chloroplast genome of *Chromera* expresses an *rff* gene, no *rff* genes were found in the apicoplast genomes of all apicomplexan parasites sequenced to date.

Annotation of the apicoplast genome of *B. microti* revealed 5 hypothetical coding sequences (*hypA–E*). The encoded proteins do not share significant homology with any protein in available databases and do not contain any recognizable functional domains. Examination of the apicoplast genomes of other apicomplexan parasites shows the presence of unknown but dissimilar proteins in the same genomic regions. Whether these CDSs are expressed or are an artifact of annotation remains to be determined.

### *B. microti* carries the minimal apicoplast genome of apicomplexa

**Figure 2:**
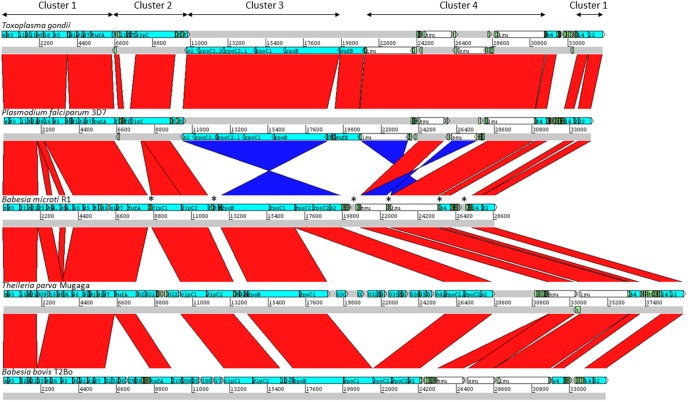
Schematic representation of gene clusters in the apicoplast genomes of various apicomplexan parasites. Comparison was performed using Mauve and BLAST analyses. The red and blue bars between chromosomal DNA sequences represent highly conserved regions in the forward and reverse directions respectively. Only highly conserved and syntenic regions were included in the present analysis. tRNA genes are marked by *.

**Figure 3:**
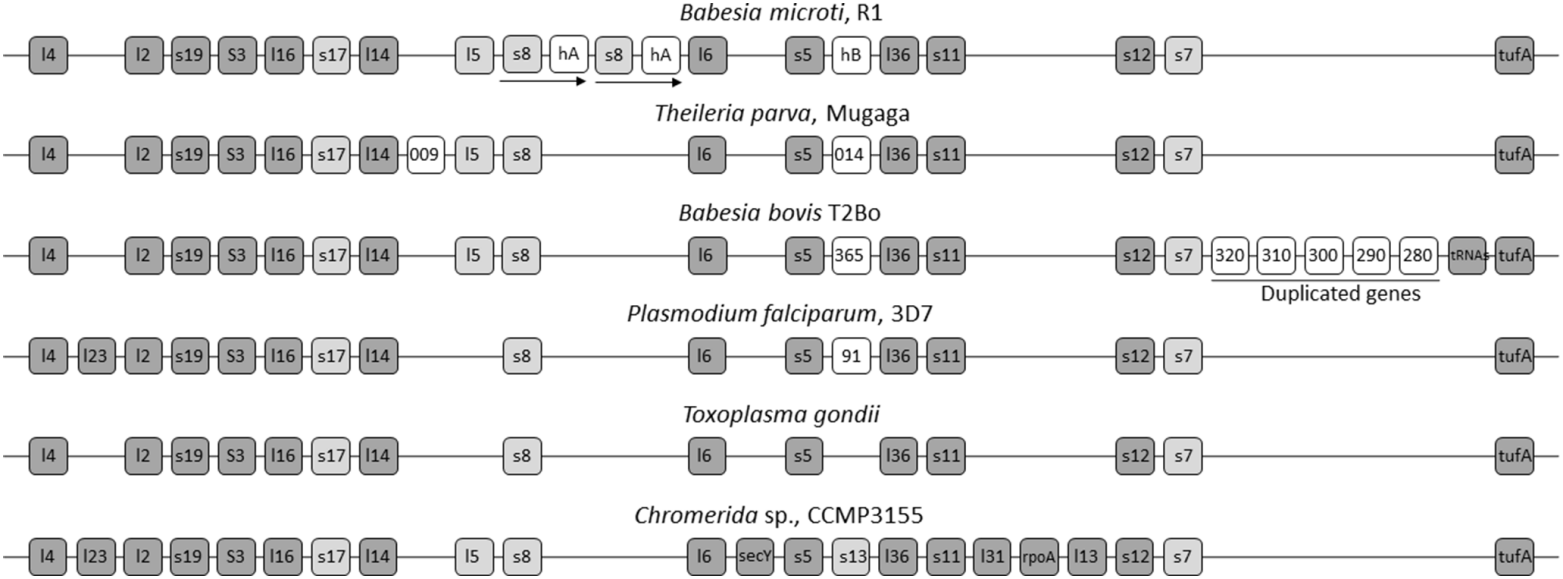
Gene organization of cluster 1 in the apicoplast genomes of *B. microti* (R1), *B. bovis* (T2Bo), *P. falciparum* (3D7), *T. gondii* and *T. parva* (Mugaga) and the chloroplast genome of *Chromera* sp. (CCMP3155). Light grey boxes represent highly divergent genes. White boxes corresponds to genes restricted to one species.

Four gene clusters in the *B. microti* apicoplast genome were found to be in a synteny with those found in other apicomplexan parasites (Fig. 2 and Figures S1 and S2 in File S1) as well as the chloroplast genomes of *Chromera* algae [27]. Cluster 1 includes genes encoding ribosomal proteins and the EF-Tu elongation factor (Fig. 3). Similar to the gene organization found in *Theileria parva*, *Toxoplasma gondii* and *Babesia bovis*, Cluster 1 of the apicoplast genome of *B. microti* lacks the *rpl23* gene. This gene is present in *Chromera* and *Plasmodium* species between *rpl2* and *rpl4* genes but was lost during the evolution of the apicoplast in most apicomplexa. Two copies of the *rps8-hypA* genes encoding S8 ribosomal protein and a hypothetic protein (Hyp-A) are found in the Cluster, whereas no *rps13*-like gene could be found in this genome. In *Chromera* sp., *rps13* is located between *rps5* and *rpl36*, whereas in most apicomplexan parasites, this region contains a gene with unknown function or lacks a CDS as in the case in *T. gondii* (Fig. 3).

**Figure 4:**
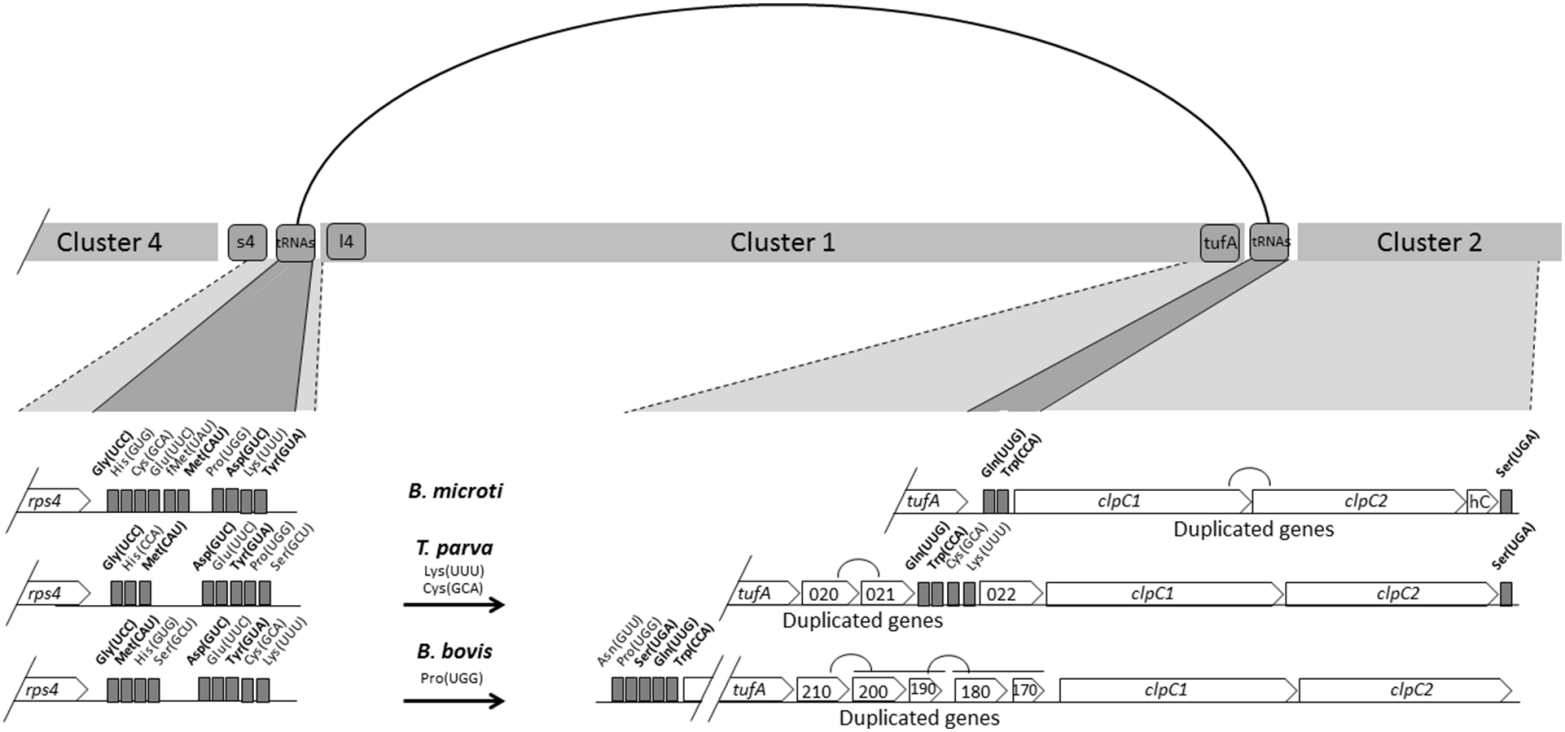
Schematic representation of the DNA regions surrounding cluster 1 in *B. microti* R1, *B. bovis* T2Bo and *T. parva* Mugaga. A line connecting the two ends of Cluster 1 indicate possible recombination events accounting for differences found in the gene organization and size between apicoplast genomes of piroplasmida.

Cluster 1 of the *B. microti* apicoplast genome is surrounded by 10 tRNA genes on the side adjacent to Cluster 4 and 2 tRNA genes for Gln(UUG) and Trp(CCA) on the side adjacent to Cluster 2 (Fig. 4). Interestingly, in *T. parva* the junction between Cluster 1 and Cluster 2 contains two more tRNA genes for Lys(UUU) and Cys(GCA), whereas in *B. bovis* this region lacks tRNA regions. Unlike *B. microti*, the junction between Cluster 1 and Cluster 2 in *T. parva* and *B. bovis* contains several putative CDSs of unknown function some of which are identical copies of the same CDS (*Tp020* and *Tp021* in *T. parva*) and (*Bb210* and *Bb200*, and cluster *Bb200*–*Bb190* and *Bb180*–*Bb170*). The duplicated CDSs found in *T. parva* do not share homology with those found in *B. bovis* (Fig. 4). Altogether, these data suggest that the region between Clusters 1 and 2 might be a hot spot of recombination, and that major recombination events involving of regions adjacent to Cluster 1 may have taken place during the evolution of piroplasmida (Fig. 4). Furthermore, the lack of duplicated genes in *B microti* may account for the differences we see in size between these organisms.

**Figure 5:**
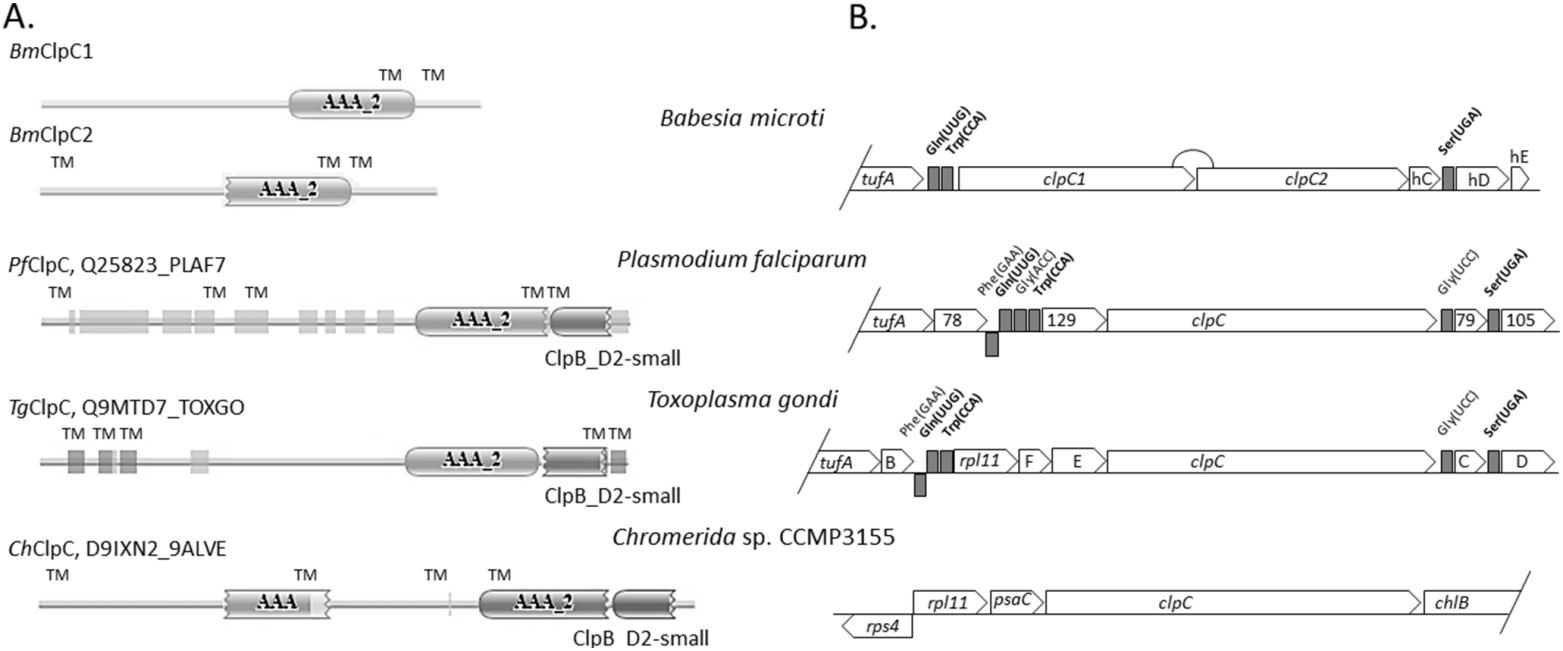
Domain structure and organization of Cluster 2 genes in *B. microti* and other apicomplexan parasites. **A.** Comparison of ClpC domain structure between *B. microti*, *P. falciparum, T. gondii* and *Chromera* sp. *B. microti* apicoplast genome encodes two ClpC proteins that lack the N-terminus part as revealed by Pfam and TMpred predictions. Other apicomplexan ClpC structures have been obtained from Pfam database using UNIPROT accession numbers. Two PfamA domains are found in ClpC proteins of apicomplexa: AAA_2 (ATPase catalytic function) and ClpB_D2-small (conserved C-terminal domain). Light grey boxes indicate regions of low complexity. Transmembrane domains were predicted by Pfam only for *T. gondii* ClpC proteins (TM). **B.** Gene organization of cluster 2. The tRNA genes of cluster 2, which are conserved in all three apicoplast genomes are in bold. Three putative genes, C, D and E present at 3′ end of *B. microti* cluster 2 have no significant homologies with each other and lack homologs in other parasites.

Cluster 2 of the *B. microti* apicoplast genome consists primarily of ClpC chaperones (Fig. 5). Similar to *B. bovis* and *T. parva*, the *ClpC* gene of *B. microti* is duplicated with both copies containing the AAA_2 ATPase domain (Fig. 4 and Fig. S3 in File S1). The region of Cluster 2 adjacent to Cluster 3 contains a Ser(UGA) tRNA and three hypothetical proteins (Hyp-C, Hyp-D and Hyp-E) (Fig. 1). The position of the Ser(UGA) tRNA is conserved in other apicomplexan parasites including *T. parva* (Fig. 4), *P. falciparum* and *T. gondii* (Fig. 5).

Similar to *B. bovis*, *P. falciparum* and *T. parva* no *rpl11* ribosomal gene was found in the *B. microti* apicoplast genome (Fig. 2 & 5). This finding suggests that either the ribosomes of these parasites do not require the L11 protein or that protein translation in the apicoplast of these parasites involves an rpl11-like gene radically divergent from that found in *T. gondii*, and prokaryotes and located on a different site in the apicoplast genome or possibly encoded by the nuclear genome. Because of the conserved gene order *rpl11*-*clpC* in *Chromera*, and *T. gondii*, the loss of the *rpl11* gene in the apicoplast genomes of parasites within the Class Aconoidasida (which includes Haemosporida and Piroplasmida) might be linked to the rearrangement of the tRNA region adjacent to the *clpC* gene.

Cluster 3 of the *B. microti* apicoplast genome includes the “RNApol cluster” and contains in addition to the RNA polymerase genes (rpoB, rpoC1, rpoC2.1 and rpoC2.2), the gene encoding the S2 ribosomal protein, *rps2* (Fig. 1 and 2). In *B. microti* as well as other apicomplexa, the alpha subunit of RNA polymerase (rpoA) gene is encoded by the nuclear genome, whereas in algae chloroplast genomes the gene encoding is present on cluster 1 (Fig. 3). Orientation of Cluster 3 genes in *B. microti*, *T. parva* and *B. bovis* is opposite to that found in *P. falciparum* and *T. gondii*, suggesting an inversion event that took place early during the evolution of piroplasmida (Fig. 2). Such an event might be responsible for the loss of the *sufB* gene in piroplasmida.

**Figure 6:**
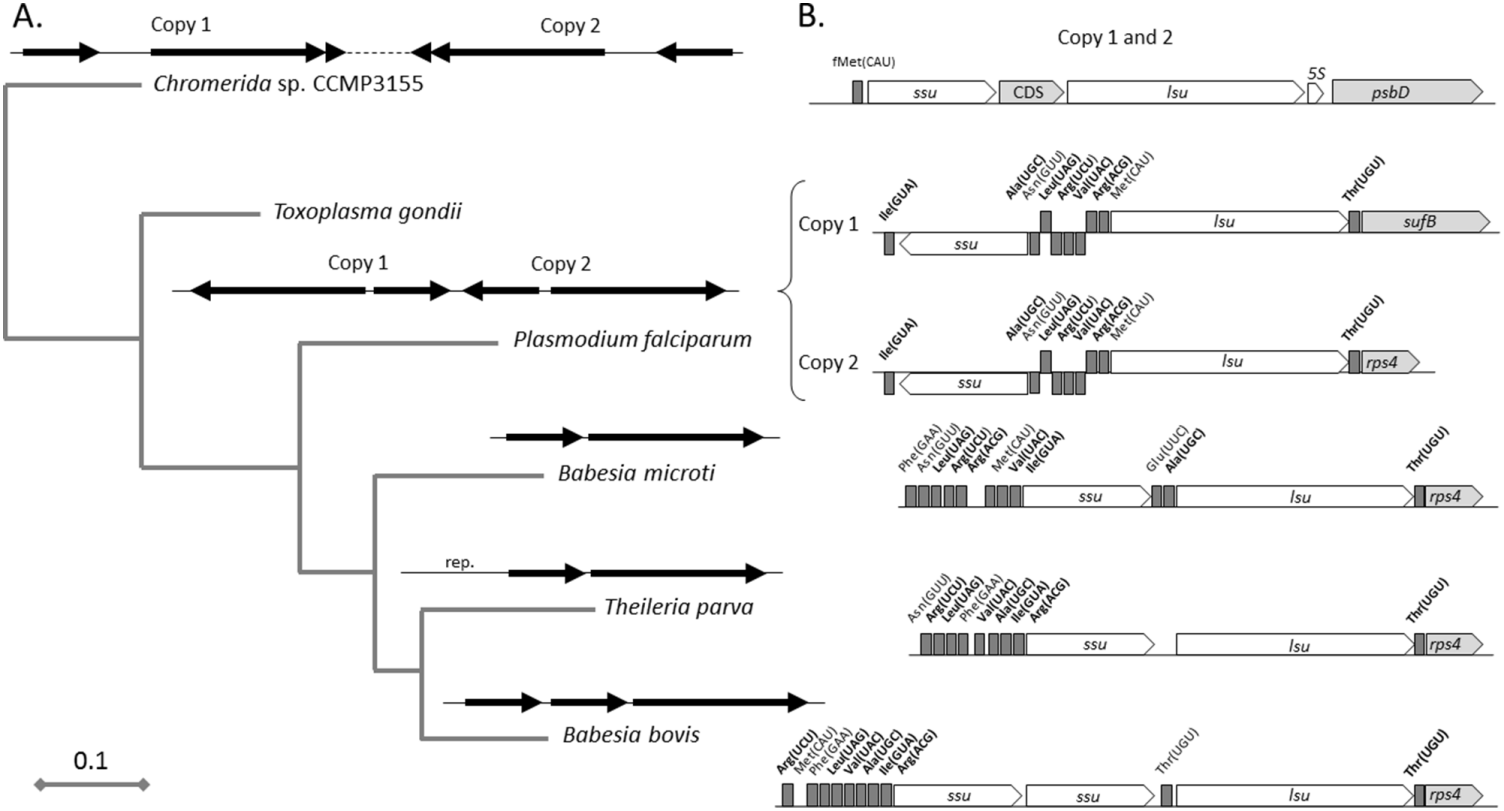
Organization and evolution of the rDNA region in the apicoplast genome of *B. microti* and other apicomplexa. **A.** Phylogenetic analysis based on *ssu* and *lsu* genes. The tree was obtained using the maximum-likelihood method with (Bootstrap over 90%). Genomic organization of rDNA regions in the apicoplast or chloroplast genomes is given on top of each branch. **B.** Gene organization of the rDNA regions. The tRNA genes that are present in all apicomplexan genomes are shown in bold. Scale bar represents the number of substitutions per site.

Cluster 4 of the apicoplast genome of *B. microti* includes rDNA genes. This region consists of a single set of *ssu* and *lsu* genes, which are transcribed in the same orientation (Fig. 6). In *Chromera* sp., *T. gondi* and *P. falciparum* apicoplast genomes, this cluster consists of two sets of *ssu* and *lsu* genes in opposite orientation (Fig. 6). Gene content and gene order in this cluster differ between species. In *Chromera*, the *ssu* and *lsu* genes are in the same orientation and separated by a CDS; in *Toxoplasma* and *Plasmodium* the *ssu* and *lsu* genes are in opposite orientation; and in *B. bovis,* 2 *ssu* genes are located upstream of the *lsu* gene and all three genes are transcribed in the same orientation. Unlike *B. microti* and *T. parva*, a second Thr(UGU) tRNA exists between the *ssu* and *lsu* genes in *B. bovis* (Fig. 6). This gene organization is likely the result of duplication events that occurred in the rDNA region during the evolution of *B. bovis*.

**Figure 7:**
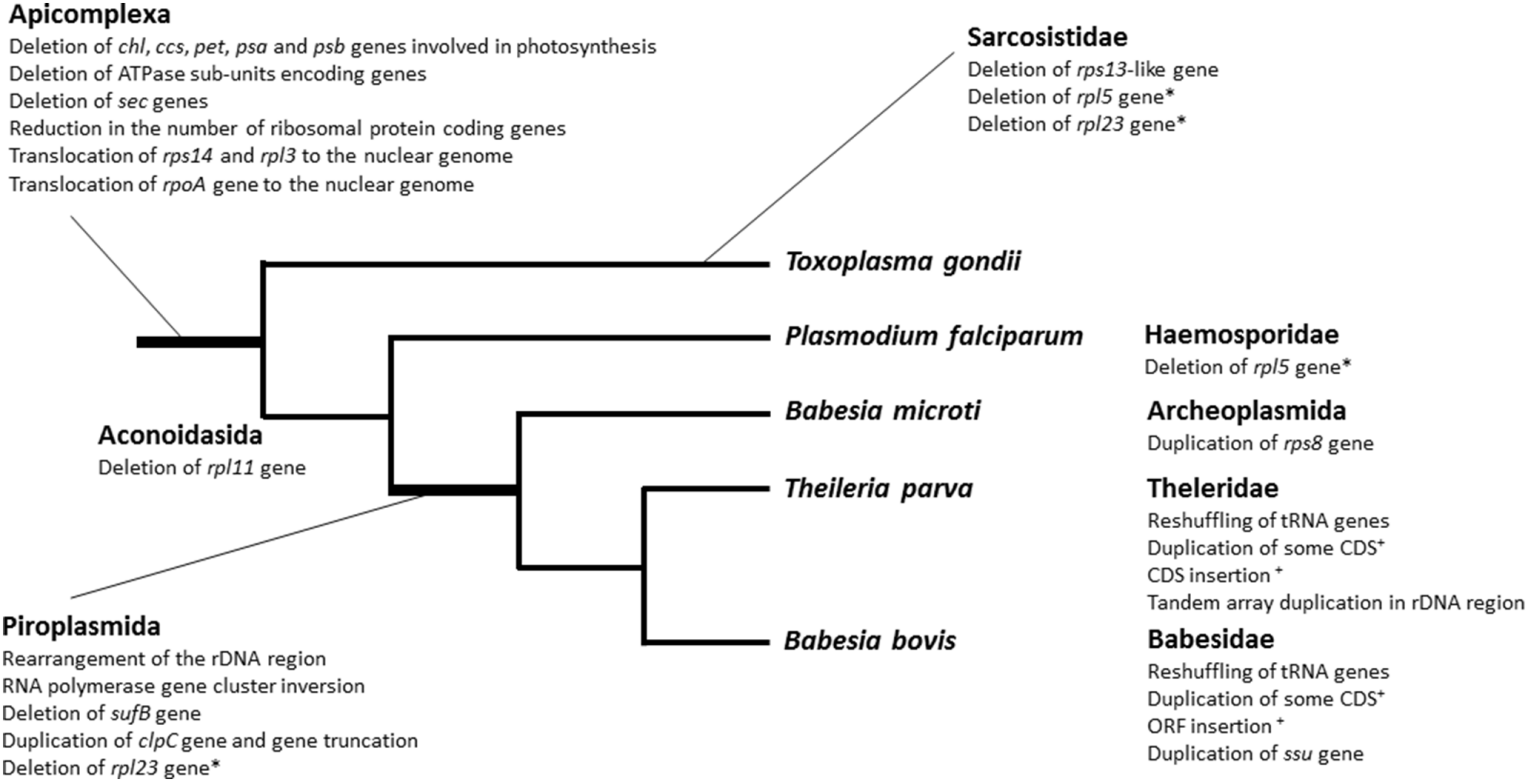
Summary of the evolution of the organization of the apicoplast genome in apicomplexan parasites. The unweighted tree was built using raw data from Figure S2 in File S1. The branch supporting the clade piroplasmida is associated with several major genomic rearrangements. *: events that occurred twice in the apicoplast evolution; +: rearrangement, duplication and insertion events observed in *B bovis* and *T. parva* involving distinct genes.

Comparison of different apicoplast genomes shows that major rearrangements took place during the various stages of apicoplast evolution (Fig. 7). While the loss of genes involved in photosynthesis represent a major early event in the evolution of the apicoplast, deletion of *sufB*, inversion of the RNApol region, reorganization of the rDNA region and duplication of the *clpC* gene represent important events that occurred during the early evolution of piroplasma. *B. microti* apicoplast genome carries these modifications but shows no DNA expansion (duplication of small regions) as is the case in *B. bovis* and *T. parva*. This suggests that *B. microti* may harbor the core apicoplast genome organization of apicomplexa.

## Conclusion

We have completed the sequencing and assembly of the apicoplast genome of *B. microti*. Our studies revealed that this 28.7 kb circular genome encodes a simple machinery devoted primarily to the transcription and translation events occurring within this organelle. The genome lacks genes associated with metabolic functions but harbors five genes encoding small hypothetical proteins whose function remains unknown. Our analysis of the apicoplast genome of *B. microti* complements our prior annotation of the nuclear genome of this pathogen, which identified several genes encoding structural and regulatory proteins and enzymes harboring an apicoplast targeting motif [11]. These nuclear encoded proteins are predicted to control important metabolic functions during the parasite life cycle in mammalian red blood cells and the tick vector. The potent activity of drugs such as azithromycin and clindamycin [28] against *B. microti* indicates that the apicoplast plays an essential role during the parasite intraerythrocytic life cycle. Targeting the apicoplast- and nuclear-encoded functions important for apicoplast maintenance and replication may help identify and design novel, potent and safer therapies for the treatment of human babesiosis.

## Supporting Information

File S1 **Figure S1:** Multiple alignment of apicoplast genomes from B. microti and other apicomplexan parasites using Mauve. Apicoplast genomes are laid out horizontally with colored blocks representing homologous regions. Gene synteny and BLAST analysis were used to define gene clusters. **Figure S2:** Major genomic rearrangements revealed by comparing apicoplast genomes of various apicomplexan parasites. Red and blue areas represent conserved syntenic regions in the forward and reverse directions, respectively. **Figure S3:** Domain structure of ClpC proteins encoded by the apicoplast genome of piroplasmida. The apicoplast genomes of the Babesia bovis T2Bo, Babesia microti R1 and Theileria parva Mugaga encode two ClpC proteins containing a conserved ATPase domain (PfamA AAA_2 domain) and several transmembrane domains predicted using TMPred (TM). The figure was generated using Pfam server either using UNIPROT accession number or by direct submission of the amino acid sequence. Regions of low complexity are represented in blue. PfamB domains are represented by horizontal lines. **Table S1:** Primers used to assemble and sequence the B. microti apicoplast genome. Coordinates are given according to the sequence available at accession number LK028575. Primer orientation: w for Watson strand and c for Crick strand.
(PDF)
