# Supplementary material for: Sequence and Annotation of the Apicoplast Genome of the Human Pathogen Babesia microti
Source: PLoS One. 2014 Oct 3;9(10):e107939. doi: 10.1371/journal.pone.0107939 (PMC4184790; doi:10.1371/journal.pone.0107939)
Supplement: File S1 — Figure S1: Multiple alignment of apicoplast genomes from B. microti and other apicomplexan parasites using Mauve. Apicoplast genomes are laid out horizontally with colored blocks representing homologous regions. Gene synteny and BLAST analysis were used to define gene clusters. Figure S2: Major genomic rearrangements revealed by comparing apicoplast genomes of various apicomplexan parasites. Red and blue areas represent conserved syntenic regions in the forward and reverse directions, respectively. Figure S3: Domain structure of ClpC proteins encoded by the apicoplast genome of piroplasmida. The apicoplast genomes of the Babesia bovis T2Bo, Babesia microti R1 and Theileria parva Mugaga encode two ClpC proteins containing a conserved ATPase domain (PfamA AAA_2 domain) and several transmembrane domains predicted using TMPred (TM). The figure was generated using Pfam server either using UNIPROT accession number or by direct submission of the amino acid sequence. Regions of low complexity are represented in blue. PfamB domains are represented by horizontal lines. Table S1: Primers used to assemble and sequence the B. microti apicoplast genome. Coordinates are given according to the sequence available at accession number LK028575. Primer orientation: w for Watson strand and c for Crick strand. (PDF) [file pone.0107939.s001.pdf]

Table S1: Primers used to assemble and sequence the *B.microti* apicoplast genome. Coordinates are given according to the sequence available at accession number LK028575. Primer orientation: w for Watson strand and c for Crick strand.

Name	Sequence	Begin	End	Strand
rps2.2	CCGATTGTTATACCATTTAACTATAAAGAAG	131	101	c
api1_tRNA	TCGAGTCTGCATCTTATTTATTTAGGTAATTAACTTAATGG	915	955	w
ctg1_ssu	GATTTAATCTCCCTTCACAAGACATTACTTTGTCTAG	1542	1506	c
BA39YPO6-R	TATAGACCCGAACCCAAATGATCT	3486	3509	w
AA3YN24-F	AGTAACGGAGGTGAACAAAGGT	5002	5023	w
AA3YN24-FF	GATTTGTAATCAGTAGAATATGAGTT	5695	5720	w
BA39YPO6-F	AACTCATATTCTACTGATTACAAATCA	5720	5694	c
AA3YN24-R	TTTTCAATAGTTAGAAAAATTCCTCATT	6967	6940	c
BA20YL13-R	AAAAGTACTAATATATTTGTTGGTGGTT	7875	7902	w
Ctg1b_F	CCACATGGAGGAGGAGAAGG	8843	8862	w
BA20YL13-RR	ATGGAGGAGGAGAAGGTAA	8847	8865	w
BA20YL13-FF	TTTTACCTTCTCCTCCTCCA	8867	8848	c
BA20YL13-LST	TAATTGAACCTTTAAGTAGTT	9616	9636	w
ctg1_rps3	TATATTCAGGTAGGTTTAATAAGTTAAATAATAGATCAAAAGTTG	9751	9795	w
BA20YL13-F	ACTTATTAAACCTACCTGAATATATA	9774	9709	c
RPL1_Long	TTATATCAAAAAGTTTTGGATCAATATCATCAAGTCAATTAGAATC	10042	10087	w
Gap3.1	TAAGTTTTTTATGGCTAAAGTTCTTTTGGTAAATAAGT	11143	11180	w
Gap3.2	AATTAATTTTATACTTATTTACCAAAAGAACTTTAGCCA	11192	11154	c
5948.1	ATAATGAAGAACGTATTAAAATGAGAAATTTTACTTTAGAAGTAGT	11705	11750	w
Gap3.3	GAAAAAATAAATATAAAAGAAAAAGTATTGACCCAATTAAGA	12127	12168	w
Gap3.4	ACTTATAAATTTTTTAATATAAGTATATATAATCTTAATTGGGT	12200	12157	c
5948.1	ATAATGAAGAACGTATTAAAATGAGAAATTTTACTTTAGAAGTAGT	12470	12515	w
Gap3.3	GAAAAAATAAATATAAAAGAAAAAGTATTGACCCAATTAAGA	12893	12933	w
Gap3.4	ACTTATAAATTTTTTAATATAAGTATATATAATCTTAATTGGGT	12965	12924	c
Gap3_PRA4	CTATAGTAAGATAATTAAAATTTAATACCCTATC	13141	13108	c
Gap3_seq1.1	AAATTATATTATTTAAAAATTACGGATTTAATAACAGT	13400	13437	w
Gap3_PRA3	CCTTTACCAGTATAAACATTATACTTTTTAATATTTTTTATTCTAC	13568	13523	c
Gap3_PRA1	GGTAATAAAACAGGATGGTGTGGATTAGGAGT	13871	13902	w
Gap3_seq1.2	ATCTCTAAATCTATTAATAAATTTAATTTTACAACCTTT	14056	14018	c
Gap3_PRA9	TTATTTTATAATTTAAAATTTTACACTTATGGTATAGGT	14303	14341	w
Gap3_PRA10	AAAAATAATAAAATATATAATTTAAAAATACCTATACCA	14370	14332	c
Gap3_seq2.1	AAATTTGTAAAAAATGTAGATTAATAAGAAGAAATAAAA	14543	14581	w
Gap3_PRA7	TTTGAATTTATAAATGATTTTGAACCACATGAAGATGAG	14824	14786	c
Gap3_PRA2	ATTATTTTAAGTTTTCTTATAATGGATGTAGAGTT	15007	15041	w
Gap3_seq2.2	TTAATTCTTTTACCTCTTATAGCTTGATTTAATGTT	15218	15183	c
Gap3_PRA8	TTAATTTTATTATTCATTTATTCCTATATTTTTTTACACCA	15577	15537	c
5929.1	TTAATGTTGTTTTACCATGATCTATATGACCTATAGTACCTACA	16270	16227	c
EFT1_long	CCTGTAACTACAGTACCTCTACCTGTTATTAAAAAACTATCTTC	16895	16852	c
AA9YN12_EFTU	TTTATAACCTGATAAAAAAGGTTTATGTCTACCTCCTTC	17190	17152	c
AA9YN12-F2	AAAATGGCTATGCCAGGTG	17263	17281	w
AA9YN12-FR1	TGTAATAATACCTGCTCC	17388	17371	c
AA9YN12-F3	AACTGAAATAGATAGGTTTG	17875	17894	w
AA9YN12-FR2	CTATTATTAGTGTTACATTGAA	18066	18045	c
AA9YN12-RR2	GTAGTTGGATTATATGTGG	18606	18624	w
AA9YN12-R3	CCTGGTGGTGCGCC	18765	18752	c
AA9YN12-RR3	GAATTTTTAAATAGAATTGATG	19061	19082	w
AA9YN12-R2	ATACTAAAGATTTACCACAAATATC	19328	19304	c
Clpc.1	TTGTTTTTTAATAATGATATTTGGATAATAGAATGTGA	19523	19560	w
Clpc.2	TATAGTATTAATAATACTTTTAAAAGAGTTATAATAATAATTAAAG	19676	19721	w
Clpc3	TTCTTTCATCATCTACAGGAGT	20379	20400	w
Gap2.1	AGCATTTGATTTGAGATCTTAGTAATATGGGTT	21142	21174	w
Gap2_seq1.1	TTAAAAATAAAAAATTATCATTAACTAAAAAAAAAA	21580	21615	w

Gap2.2	TTTTGTTGAATTTATTTTTATTGAATATGTTTTATT	22125	22090	c
rpoB3	GTTAAATATGTTATTGTATTTGT	22298	22276	c
Gap2_seq1.2	TTTTAAAATAATTTCACTTATTGAAGAGTGTTT	22467	22435	c
rpoB.1	ATGCATTTTTGAACCCATAAGACCCCTAC	23004	22976	c
rpoB.2	TTTATTACAATACCAGATGAATAGGAAATTATACAA	23123	23088	c
rpoC.2	TATTAGTTGGTTATCTTAATTTAGCTATTATACATG	25725	25760	w
rpoC.1	GACATATAATATATACTGTAATTCATTAAATTCTGAAC	25822	25859	w
8452.1	AAAAAAAAGTCAATTTATACAAATATCACAAT	26221	26252	w
Gap1.2	AATATTATAAAAAAAATAGATAGGGATGATTTTGA	27201	27235	w
Gap_1.1	ATATATTGTTGAGTATTAGTATTTAATAATAAACCA	27325	27290	c
rps2.1	AATTATAAAATAATCTATACCATCCAGATCCT	27963	27932	c
api1_rps2	CACTTTTCCATGAAGATAAAAAATAAAAGTTTAATGTTAATTCTG	28265	28221	c

Figure S1

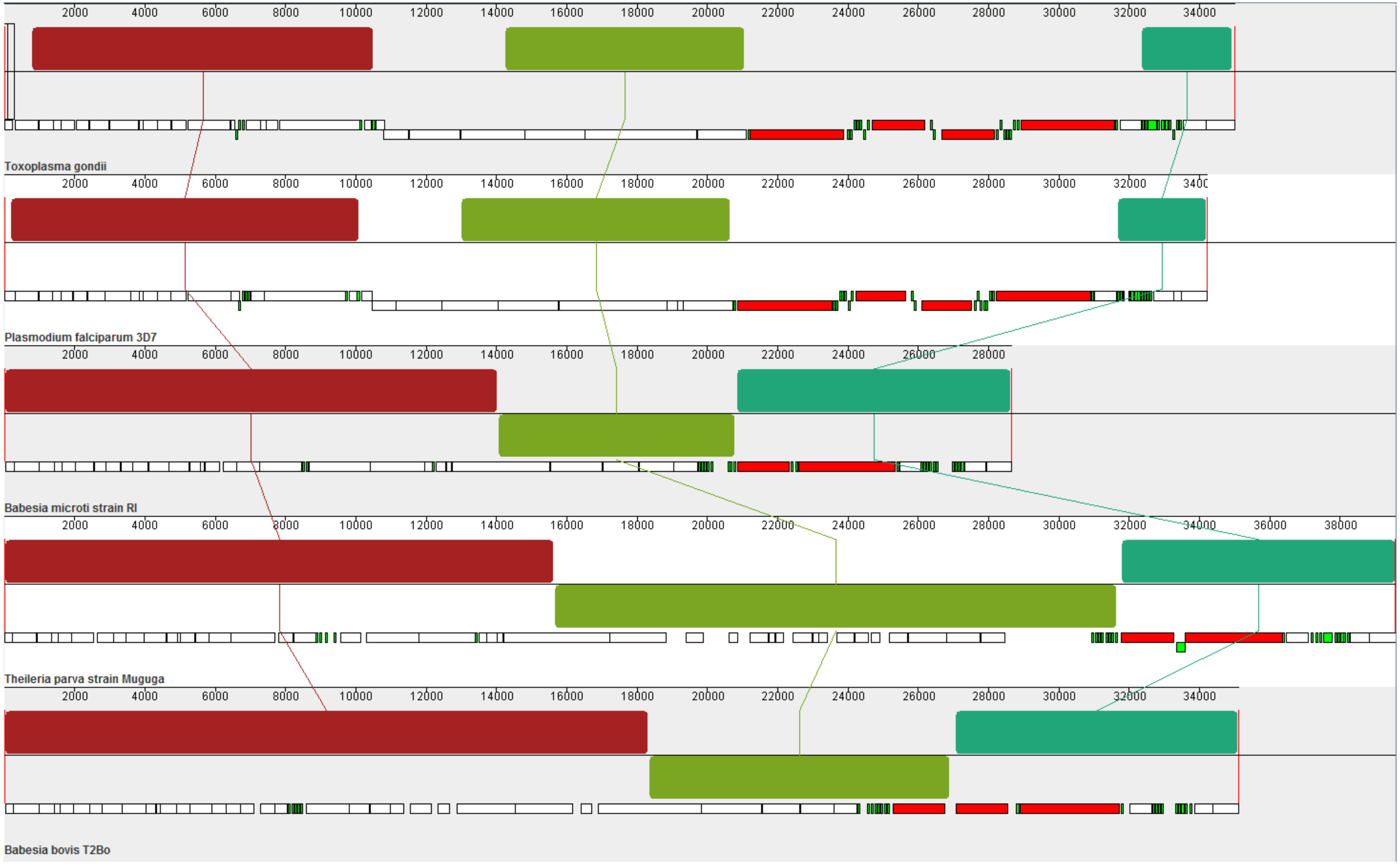

Toxoplasma gondii
Plasmodium falciparum 3D7
Babesia microti strain RI
Theileria parva strain Muguga
Babesia bovis T2Bo

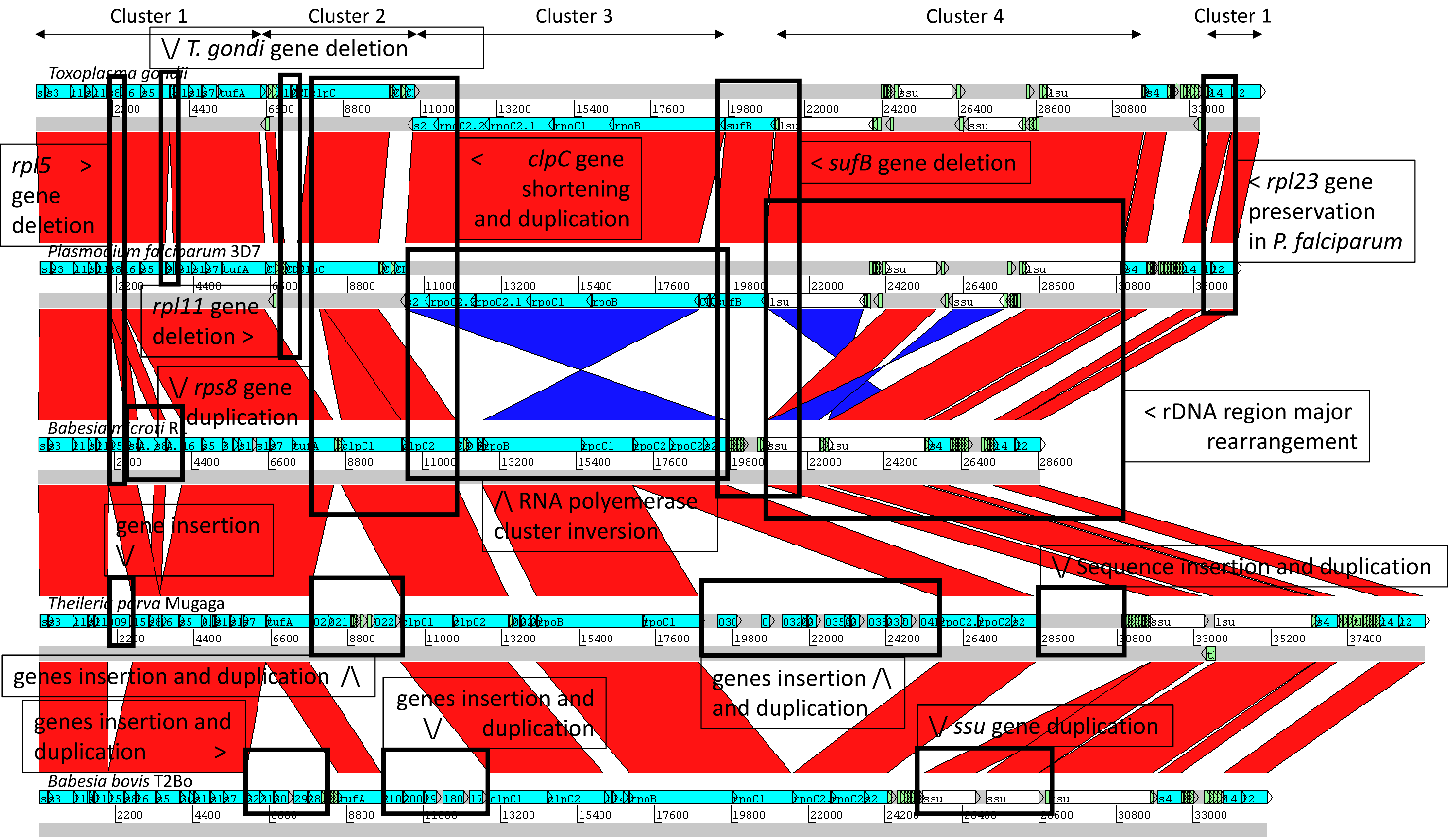
Cluster 1
Cluster 2
Cluster 3
Cluster 4
Cluster 1
\/ T. gondi gene deletion
Toxoplasma gondii
rpl5 > gene deletion
< clpC gene shortening and duplication
< sufB gene deletion
< rpl23 gene preservation in P. falciparum
Plasmodium falciparum 3D7
rpl11 gene deletion >
\/ rps8 gene duplication
Babesia microti RI
< rDNA region major rearrangement
/\ RNA polyemerase cluster inversion
gene insertion \/
\/ Sequence insertion and duplication
Theileria parva Mugaga
genes insertion and duplication /\
genes insertion and duplication >
genes insertion and \/ duplication
genes insertion /\ and duplication
\/ ssu gene duplication
Babesia bovis T2Bo

Figure S2

Figure S3

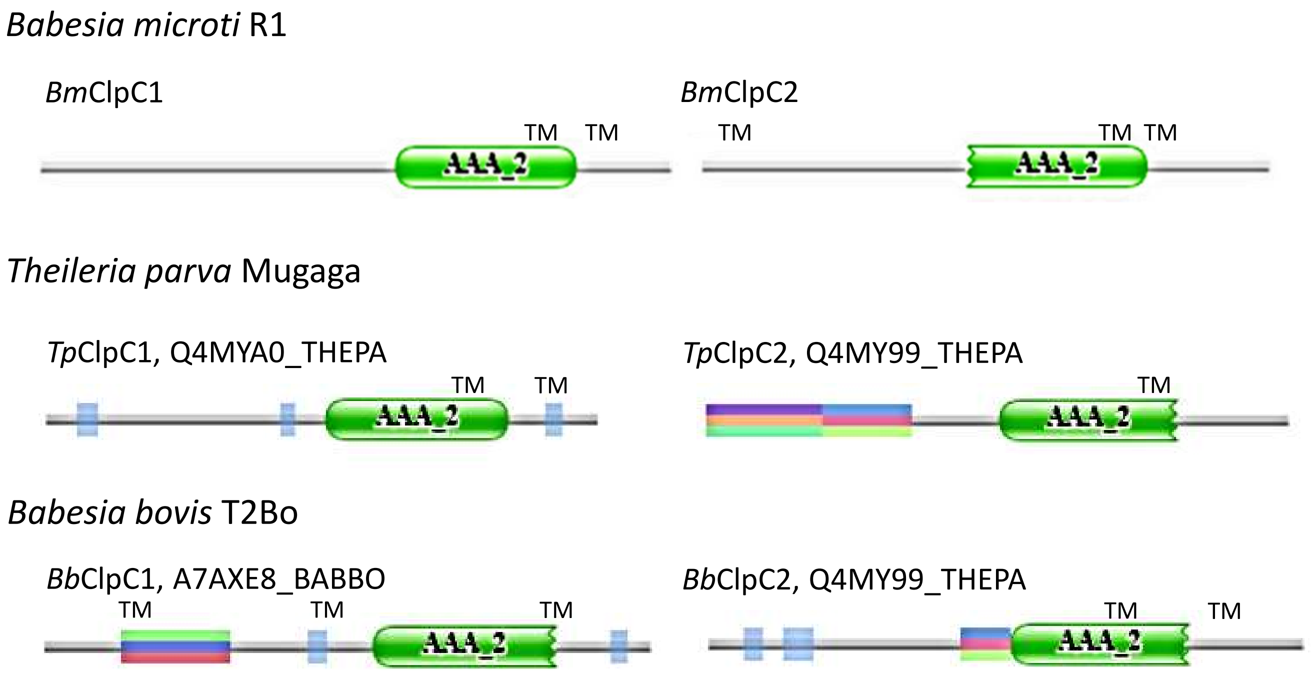
Babesia microti R1
*Bm*ClpC1
TM TM
AAA_2
*Bm*ClpC2
TM
TM TM
AAA_2
Theileria parva Mugaga
*Tp*ClpC1, Q4MYA0_THEPA
TM TM
AAA_2
*Tp*ClpC2, Q4MY99_THEPA
TM
AAA_2
Babesia bovis T2Bo
*Bb*ClpC1, A7AXE8_BABBO
TM TM TM
AAA_2
*Bb*ClpC2, Q4MY99_THEPA
TM TM
AAA_2
